# Optimizing the Orientation of a Suture Button to Stabilize the Distal Radioulnar Joint in a Sawbones Model

**DOI:** 10.1016/j.jhsg.2022.06.007

**Published:** 2022-08-26

**Authors:** Makoa Mau, John Livingstone, Gordon Lee, Patrick Murray

**Affiliations:** ∗ John A Burns School of Medicine, University of Hawai'i, Honolulu, HI; † Department of Orthopedic Surgery, University of Hawai'i, Honolulu, HI

**Keywords:** Distal oblique bundle, Distal radioulnar joint, Instability, Stabilization, Suture buttons

## Abstract

**Purpose:**

When left untreated, distal radioulnar joint (DRUJ) instability leads to prolonged wrist pain and weakness during pronosupination. Current treatment options are technically demanding and result in mixed outcomes. This study used a synthetic bone model to evaluate the potential of using a suture button to stabilize the DRUJ and find its optimal configuration*.*

**Methods:**

A suture button was placed between the radius and ulna of a synthetic bone model with DRUJ instability. The suture button was placed straight across or in an oblique orientation while the forearm was in 60° of pronation, neutral, or 60° of supination for 6 configurations. The range of motion, dislocation events, dorsal translation, volar translation, and gaps between the radius and ulna were measured and compared among these 6 configurations.

**Results:**

Full range of motion (ROM) was achieved in all configurations except for suture buttons placed while the forearm was in 60° pronation. Obliquely placed suture buttons led to more dislocations than straight across suture buttons. The 2 configurations that offered full ROM with the greatest stability were straight across 60° supination and straight across neutral configuration, with the supinated configuration slightly improving stability.

**Conclusions:**

In this model, suture buttons restored DRUJ stability while maintaining full ROM, indicating that suture buttons have the potential to be used as a treatment option for stabilization of DRUJ. The optimal configuration of a suture button is likely in the straight across 60° supinated configuration, as it provides the greatest stability without sacrificing ROM compared with the other suture button configurations.

**Clinical relevance:**

Additional treatment options for the stabilization of DRUJ are needed. Suture buttons may be of use.

Distal radioulnar joint (DRUJ) instability has traditionally been associated with Galeazzi fractures of the forearm. However, recent studies have found that DRUJ instability also occurs with common distal radius fractures, with an incidence between 7% and 35%.^1^, ^2^, ^3^ Other causes of DRUJ instability include injuries to the triangular fibrocartilage complex and Essex-Lopresti injuries.^1^^,^^4^ Although acute pain associated with these injuries often subsides over time, chronic DRUJ instability can cause ulnar-sided wrist pain and limited pronosupination.^5^

Treatment of DRUJ instability can include repair of the triangular fibrocartilage complex, reconstruction of the DRUJ, or immobilization of the wrist and forearm with or without percutaneous pinning.^6^^,^^7^ The triangular fibrocartilage complex is not always reparable, especially in chronic tears with ligamentous retraction.^8^ In these cases, the DRUJ can be reconstructed with an extrinsic radioulnar tether, ulnocarpal sling, or reconstruction of the volar and dorsal distal radioulnar ligaments (DRULs).^9^ These procedures are technically demanding, and many surgeons have opted to repair the distal oblique bundle (DOB)^10^ of the interosseous membrane instead.^11^^,^^12^ Immobilization with or without percutaneous pinning may be used in conjunction with any of these procedures or in isolation.^6^^,^^13^^,^^14^ Although immobilization can lead to excellent functional outcomes, patients often have a reduced total rotational arc of motion.^15^

Using a suture button to reconstruct the DRUJ has been shown to stabilize the DRUJ and mimic natural pronosupination in cadaveric studies^16^ and several case reports.^17^^,^^18^ Although using a suture button is a potential solution for DRUJ instability, the optimal configuration for a suture button has not been elucidated. The purpose of this study was to compare the stability and range of motion (ROM) provided by a suture button in an unstable DRUJ bone model when placed in various configurations. The primary outcomes of this study are the ROM and stability of the DRUJ after being stabilized with a suture button construct. The hypothesis of this study was that a neutral straight across suture button configuration would allow full ROM and provide the most stability for any of the suture button configurations tested. Although preliminary, this study is a starting point for future cadaveric studies and clinical investigations.

## Materials and Methods

**Figure 1:**
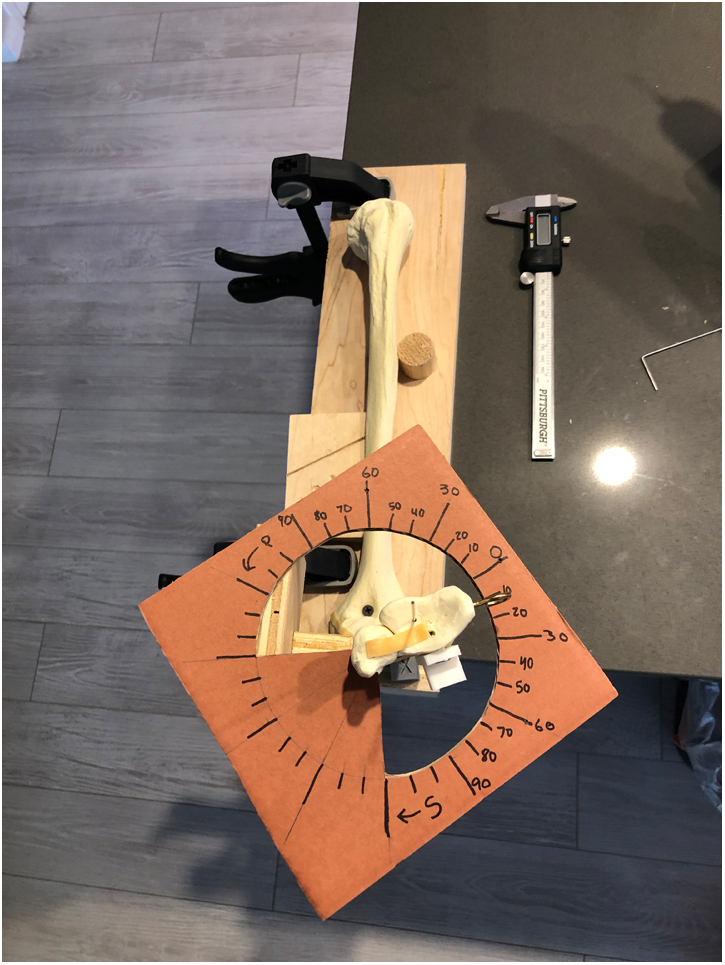
The Sawbones articulated elbow model, jig, and protractor used in this study. The ulna was anchored to the jig, while the radius was allowed to freely pronosupinate. The DRUJ was made unstable by removing the elastic band around the DRUJ, while proximal radioulnar joint elastic band was left intact. A protractor was placed immediately proximal to the DRUJ and fixed to the ulna, with the center of the protractor aligned to the center in the axial plane.

A Sawbones articulated elbow model was used for this study (SKU:1024-1). The model was mounted into a jig to secure the ulna firmly while allowing full pronosupination of the radius. A protractor was placed immediately proximal to the DRUJ and fixed to the ulna with the center of the protractor aligned to the center of the ulna in the axial plane (Fig. 1). The neutral position of the forearm was determined by finding the maximum amount of pronation and supination of the model, and splitting this total ROM in half. The DRUJ was made unstable by removing the elastic band, which wraps around the ulna and secures it to the radius. The proximal radioulnar joint elastic band was left intact.

**Figure 2:**
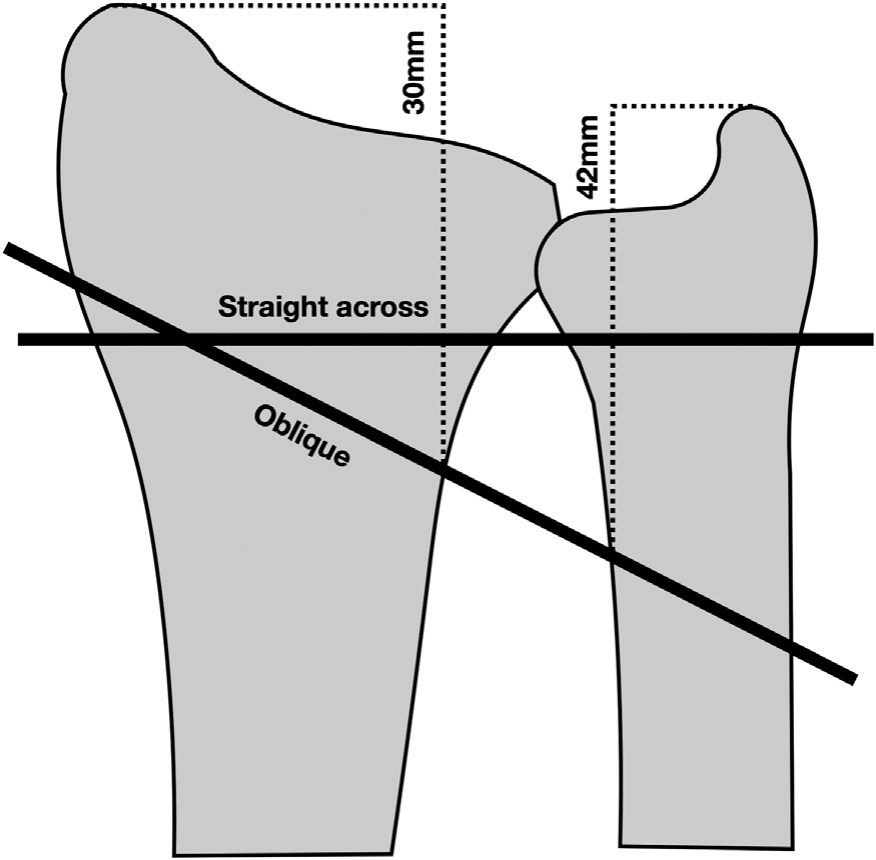
Illustration of the oblique and straight across configurations. The oblique configuration replicated the anatomy of the DOB with the interosseous portion of the suture button between 30 mm proximal to the radial styloid and 42 mm proximal to the ulnar styloid. The straight across configuration of the suture button was created by drilling a single drill hole from the ulna to the radius, proximal to the DRUJ and perpendicular to the ulna.

Suture buttons were placed straight across the radius and the ulna or in an oblique orientation. The oblique orientation of the suture button was based on the anatomy of the DOB with the interosseous portion of the suture button between 30 mm proximal to the radial styloid and 42 mm proximal to the ulnar styloid (Fig. 2).^19^ The straight across the configuration of the suture button was created by drilling a single drill hole just proximal to the DRUJ and perpendicular to the ulna (Fig. 2). In both configurations, the suture button was passed through the radius and ulna when the forearm was in 3 positions: 60° supination, neutral, and 60° pronation. The suture button used for this study was the Arthrex Mini TightRope Repair Kit (Arthrex Inc). The bone tunnel for the suture button was drilled through the radius and ulna using a 2.5 mm drill bit. The suture button was passed through the tunnel with a Kirschner wire and a suture tail. Once the buttons were engaged, the suture button was tensioned to 20 N with a calibrated spring force gauge and clamped with a hemostat. There is no literature to support a specific suture button tension, and the authors felt that 20 N of tension was appropriate based on clinical experience. The hemostat was used to clamp the suture button to allow the suture button to be reused for multiple configurations.

**Figure 3:**
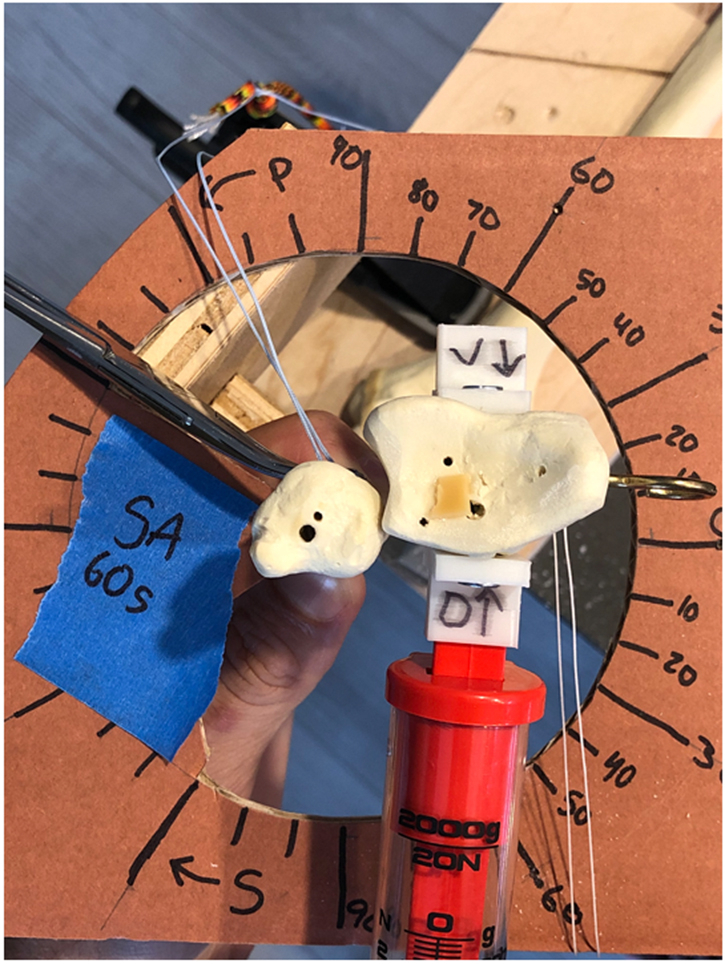
Testing of dorsal and volar translation of the ulna in the straight across 60° supinated configuration. The radius was translated volarly and dorsally, respectively, with a spring force gauge using 20 N. The force gauge was secured with a custom three-dimensional printed bracket screwed into the radius. The bracket allowed perpendicular force in the radius, which prevented unwarranted rotation. The eye hook in the radial styloid was used to test gapping.

ROM, dorsal translation, volar translation, and gapping were tested in 6 different suture button configurations: oblique orientation in 60° supination, 60° pronation, and neutral, and straight across orientation in 60° supination, 60° pronation, and neutral. The radius was rotated around the fixed ulna to test the ROM, and the force required to achieve 90° supination and 90° pronation was noted. It was noted that full ROM was not achievable with 20 N of force. Force was measured with a spring force gauge attached to an eye hook placed in the radial styloid. The force gauge was pulled perpendicular to the eye hook throughout ROM testing. With a spring force gauge using 20 N, the radius was translated volarly and dorsally to test the dorsal and volar translation of the ulna, respectively.^11^ The force gauge was secured with a custom three-dimension printed bracket screwed into the radius. The custom three-dimensional printed bracket allowed the force to be transmitted perpendicular to the radius, preventing the radius from rotating during translation (Fig. 3). The same eye hook used for ROM testing was pulled at 20 N with the force gauge to test gapping.

**Figure 4:**
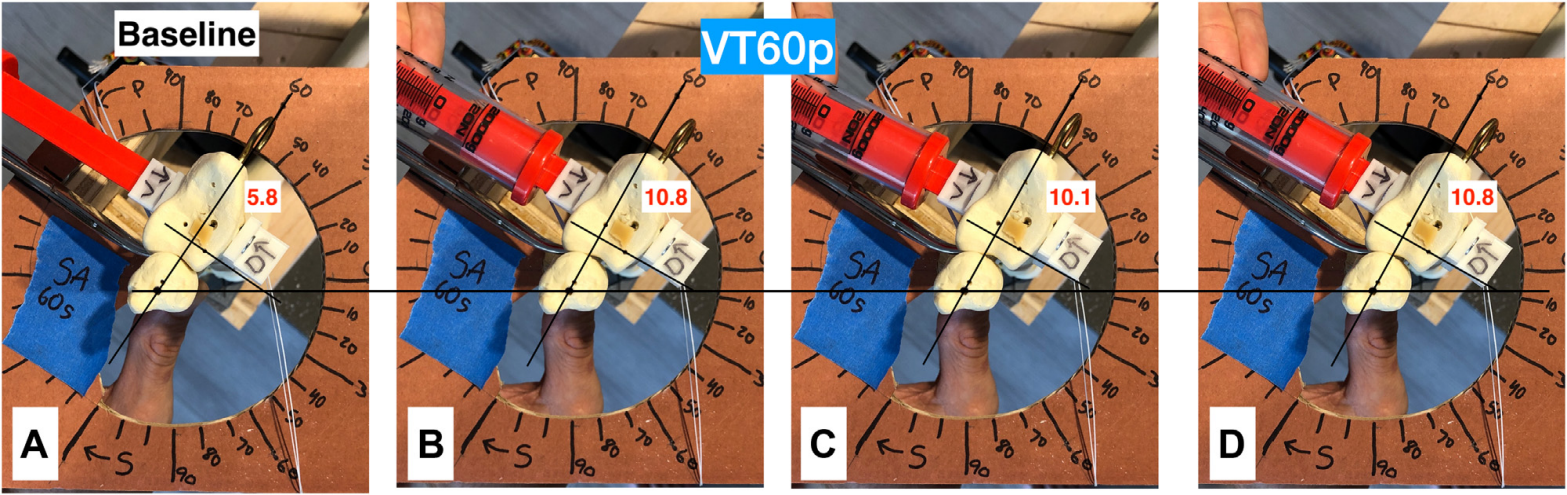
ImageJ analysis of photos taken measuring volar translation at 60° pronation in the straight across 60° supinated configuration. Figure 4A demonstrates the baseline photo (no force applied to the radius). Figure 4B, C, and D were taken with an applied 20 N force. The red device seen in the top left of each photo is the spring force gauge. The numbers shown in red are the distance between an angled line at 60° and a small hole drilled in the radius, just below and to the right of this angled line.

Photos were taken along the axial plane of the radius and ulna with an iPhone X securely mounted to a tripod to measure gapping and translation of the ulna and radius. Baseline photos without any forces applied to the setup were taken at neutral, 60° of pronation, and 60° of supination before testing. Each suture button orientation was tested for 3 movements (gapping, dorsal translation, and volar translation) at 3 forearm positions (neutral, 60° supination, and 60° pronation). Each testing scenario was repeated 3 times and averaged to reduce the effect of outliers. There were 30 individual photos collected for each of the 6 suture button configurations for 180 photos. These photos were analyzed with the freely available ImageJ software. All photos were uniformly scaled before taking measurements. The images for each motion tested were first aligned with the baseline photo. This was done by drawing a horizontal line from a marker on the ulna of the baseline photo and then aligning the remaining photos to that line (Fig. 4). An angled line was drawn from the marker on the ulna at the angle of the motion being tested. For the example in Figure 4, this was 60° of pronation. A perpendicular line was drawn from the marker on the radius to this angled line, and the distance between them was measured. The baseline photo measured 5.8 mm, whereas the volar translation photos measured 10.8 mm, 10.1 mm, and 10.8 mm. The difference between the translation and baseline measurements was used to calculate the amount of volar translation. This was repeated for dorsal translation and gapping as well. This method of measuring translation is similar to other published literature.^11^^,^^20^^,^^21^

## Results

The force needed to achieve full ROM and the dislocation positions are presented in Table 1. Placing the suture button while the forearm was in 60° of pronation led to a loss of supination in both the straight across and oblique orientations. All other configurations led to at least one dislocation event, defined as a complete translation of the ulna from the radioulnar joint. The most common position for dislocation was the dorsal translation in 60° of supination. The straight across neutral and supinated configurations had only one dislocation event, whereas the oblique neutral and supinated configurations had 2 dislocation events.

**Table 1:** The Average Force Needed to Achieve Full ROM and Dislocation Positions∗∗

	Straight across: neutral	Straight across: 60° p	Straight across: 60° s	Oblique: neutral	Oblique: 60° p	Oblique: 60° s
Force (N) to achieve 90° supination	1.08 (0.29)	>20 N	0	0	5.33∗ (0.29)	0
Force (N) to achieve 90° pronation	8.17 (0.58)	0	2.67 (0.14)	1.5 (0.00)	0	9.33 (0.29)
Dislocation positions	D60° s		D60° s	D60° s D0°		D60° s V60° s

D, dorsal; p, pronation; s, supination; V, volar.

The gray columns represent the configurations where full ROM was not achievable.

∗ In the oblique 60° p configuration, supination led to bony impingement and an incongruous DRUJ.

∗∗ When possible, the standard deviation is given in parentheses.

The dorsal translation, volar translation, and gapping in each configuration, as well as their cumulative averages among all configurations, are presented in Table 2. The greatest dorsal translation and gapping were in the oblique neutral position, and the greatest volar translation was in the oblique 60° supinated configuration. If these unstable configurations are excluded along with the 2 configurations which did not permit full ROM (the pronated configurations), the only remaining configurations are the straight across neutral and 60° supinated configurations. Between these 2 configurations, the straight across 60° supinated configuration had the least amount of dorsal translation, gapping, and volar translation.

**Table 2:** Average DT, Average VT, and Average Gapping (Gap) in Each Configuration and the Average DT, VT, and Gap Among All Configurations∗∗∗

	Straight across: neutral	Straight across: 60° p	Straight across: 60° s	Oblique: neutral	Oblique: 60° p	Oblique: 60° s
DT at 0°	3.73 (0.75)	1.03 (0.35)	5.57 (0.42)	19.50 (0.36)	4.33 (0.47)	0.43 (0.58)
Gap at 0°	1.17 (0.06)	0.17 (0.12)	1.20 (0.71)	4.60 (0.20)	0.97 (0.35)	0.60 (0.10)
VT at 0°	2.50 (1.11)	2.00 (0.00)	2.17 (0.55)	4.83 (0.45)	0.90 (0.35)	4.07 (0.47)
DT at 60° p	2.37 (0.23)	4.13 (0.70)	0.73 (0.38)	8.10 (0.26)	9.77 (1.10)	0.73 (0.51)
Gap at 60° p	0.70 (0.44)	1.07 (0.15)	0.30 (0.14)	4.23 (0.15)	1.90 (0.46)	0.33 (0.06)
VT at 60° p	1.53 (1.27)	1.27 (0.25)	4.77 (0.40)	4.77 (0.25)	2.90 (1.15)	0.87 (0.31)
DT at 60° s	14.50 (0.26)	0.83 (0.15)	13.47 (0.06)	22.30 (0.10)	7.60 (0.56)	13.37 (0.61)
Gap at 60° s	0.57 (0.06)	0.07 (0.06)	0.40 (0.28)	6.23 (0.38)	0.30 (0.17)	6.90 (0.44)
VT at 60° s	6.73 (0.32)	2.20 (0.17)	3.37 (0.72)	6.53 (0.06)	0.00	20.00
Average DT	6.87 (0.17)	2.00 (0.40)	6.59 (0.15)	16.63 (0.23)	7.23 (0.38)	4.84 (0.54)
Average Gap	0.81 (0.32)	0.44 (0.55)	0.63 (0.49)	5.02 (1.06)	1.06 (0.80)	2.61 (3.72)
Average VT	3.59 (2.76)	1.82 (0.49)	3.44 (1.30)	5.38 (0.99)	1.27	8.31

DT, dorsal translation; P, pronation; S, supination; VT, volar translation.

The gray columns highlight the highest value among the 6 configurations per row.

∗ Each measurement was repeated 3 times during testing. The values presented in this table are the average of these 3 measurements.

∗∗ When possible, the standard deviation is given in parentheses.

## Discussion

This study suggests that a straight across and supinated suture button configuration is the optimal orientation for restoring DRUJ stability. The oblique orientation was less stable than the straight across orientation. Several studies have found that less than half of individuals have a DOB.^11^^,^^19^^,^^22^ This does not answer how much the DOB contributes to overall DRUJ stability in the average patient. Other studies have demonstrated that the interosseous membrane is slack in pronation, and the distal interosseous membrane is strained in supination.^23^ This causes the trajectory of the DOB to be suboptimal since, ideally, the suture button would maintain constant tension throughout pronosupination.

On the other hand, the straight across orientation was chosen to mimic the DRUL. The DRUL dynamically stabilizes the DRUJ as the dorsal DRUL tightens during pronation while the volar DRUL tightens during supination.^24^ The authors of this study postulate that the straight across orientation, which places the suture button approximately between the dorsal and volar DRUL, may provide the most stability throughout pronosupination and be less likely to lose tension than the oblique orientation.

The main limitation of this study was the unvalidated testing method using a synthetic bone model. Although the model replicates the basic mechanics of the DRUJ, it does not mimic normal translation, gapping, and the ROM one would expect from repeating this study on a cadaveric model. Furthermore, this study did not incorporate the changes in ulnar variance seen with natural pronosupination, which may have introduced unforeseen changes in measurements.^25^ In this model, the relative motion of the DRUJ in this model closely resembles in-vivo motion, and the bony anatomy is identical. The pronosupination and translation of the model, when stabilized with the optimal suture button configurations, were similar to in-vivo measurements from other studies.^20^^,^^21^ However, this does not obviate the need for further testing. Although the synthetic bones used in this study are not validated as a DRUJ model, they allowed the authors to determine whether a suture button should be considered a treatment option and a topic for future cadaveric studies without using important resources and research funds.

This study has several strengths. First, all motions were measured precisely with ImageJ software, enabling appropriate photo scaling and application of uniform digital markers, thus reducing variability. Second, the amount of tension used when placing the suture button, when testing translation and gapping, and when testing ROM was standardized. As mentioned previously, the suture button was tensioned to 20 N, a force that the authors felt appropriate based on clinical experience. If the suture button was tensioned with greater force, it is likely that the amount of translation, gapping, and ROM would decrease. The authors hypothesized that this would likely affect all configurations equally and not change the conclusions of this study. There was no notable change in translation or gapping with repetitive testing, suggesting that the hemostat was maintaining suture button tension throughout testing. The force chosen for dorsal and volar translation was based on previous cadaveric studies to replicate normal physiologic loading.^11^ Finally, the potential impact of outliers was reduced by averaging multiple measurements of each movement at the various configurations.

Future studies will focus on repeating these methods on a cadaveric model. Reproduction in cadavers should allow for greater mimicry of normal physiologic loading. Computed tomography and axial imaging of the DRUJ can also assess the DRUJ for a concentric reduction after suture button placement. Future studies should account for the change in ulnar variance identified in pronosupination and compare the longevity of the suture button construct to DOB reconstruction with cyclic loading.^25^^,^^11^ Anatomic studies may aid in identifying potential suture button procedural complications, such as tendon irritation to the extensor carpi ulnaris.

In conclusion, this study suggests that suture buttons have the potential to stabilize the DRUJ and maintain pronosupination when placed in a straight across orientation in either a neutral or 60° supination position. This study suggests that placing a suture button construct when the forearm is in a pronated position will probably prevent full supination. If used clinically, this construct may provide patients with early ROM and stability without needing immobilization or DRUJ reconstruction. Future cadaveric studies will be required to validate this promising technique.
